# Mild traumatic brain injury affects the features of migraine

**DOI:** 10.1186/s10194-021-01291-x

**Published:** 2021-07-22

**Authors:** Ryotaro Ishii, Todd J. Schwedt, Meesha Trivedi, Gina Dumkrieger, Melissa M. Cortez, K. C. Brennan, Kathleen Digre, David W. Dodick

**Affiliations:** 1 Department of Neurology, Mayo Clinic Arizona, Phoenix, Arizona USA; 2 Department of Neurology, Kyoto Prefectural University of Medicine, Kyoto, Japan; 3 Department of Neurology, University of Utah, Salt Lake City, Utah USA

**Keywords:** Migraine, Mild traumatic brain injury (mTBI), Disability, Post-traumatic headache, Physical abuse, Psychiatric comorbidity, Depression, Anxiety, Chronic migraine, American registry for migraine research (ARMR)

## Abstract

**Background:**

Headache is one of the most common symptoms after concussion, and mild traumatic brain injury (mTBI) is a risk factor for chronic migraine (CM). However, there remains a paucity of data regarding the impact of mTBI on migraine-related symptoms and clinical course.

**Methods:**

Of 2161 migraine patients who participated in the American Registry for Migraine Research between February 2016 and March 2020, 1098 completed questions assessing history of TBI (50.8%). Forty-four patients reported a history of moderate to severe TBI, 413 patients reported a history of mTBI. Patients’ demographics, headache symptoms and triggers, history of physical abuse, allodynia symptoms (ASC-12), migraine disability (MIDAS), depression (PHQ-2), and anxiety (GAD-7) were compared between migraine groups with (*n* = 413) and without (*n* = 641) a history of mTBI. Either the chi-square-test or Fisher’s exact test, as appropriate, was used for the analyses of categorical variables. The Mann-Whitney test was used for the analyses of continuous variables. Logistic regression models were used to compare variables of interest while adjusting for age, gender, and CM.

**Results:**

A significantly higher proportion of patients with mTBI had CM (74.3% [307/413] vs. 65.8% [422/641], *P* = 0.004), had never been married or were divorced (36.6% [147/402] vs. 29.4% [187/636], *P* = 0.007), self-reported a history of physical abuse (24.3% [84/345] vs. 14.3% [70/491], *P* <  0.001), had mild to severe anxiety (50.5% [205/406] vs. 41.0% [258/630], *P* = 0.003), had headache-related vertigo (23.0% [95/413] vs. 15.9% [102/640], *P* = 0.009), and difficulty finding words (43.0% [174/405] vs. 32.9% [208/633], *P* <  0.001) in more than half their attacks, and headaches triggered by lack of sleep (39.4% [155/393] vs. 32.6% [198/607], *P* = 0.018) and reading (6.6% [26/393] vs. 3.0% [18/607], *P* = 0.016), compared to patients without mTBI. Patients with mTBI had significantly greater ASC-12 scores (median [interquartile range]; 5 [1–9] vs. 4 [1–7], *P* < 0.001), MIDAS scores (42 [18–85] vs. 34.5 [15–72], *P* = 0.034), and PHQ-2 scores (1 [0–2] vs. 1 [0–2], *P* = 0.012).

**Conclusion:**

Patients with a history of mTBI are more likely to have a self-reported a history of physical abuse, vertigo, and allodynia during headache attacks, headaches triggered by lack of sleep and reading, greater headache burden and headache disability, and symptoms of anxiety and depression. This study suggests that a history of mTBI is associated with the phenotype, burden, clinical course, and associated comorbid diseases in patients with migraine, and highlights the importance of inquiring about a lifetime history of mTBI in patients being evaluated for migraine.

## Background

Traumatic brain injury (TBI) results from an external mechanical force to the brain [1]. The United States Centers for Disease Control and Prevention estimates that 1.4–3.8 million TBIs occur each year in the United States, with the majority being considered “mild” in severity [2]. According to the International Classification of Headache Disorders 3rd edition (ICHD-3), post-traumatic headache (PTH) is defined as headache that begins or substantially worsens within 7 days of a trauma or injury to the head and/or neck [3]. Persistent PTH (i.e. PTH that has been present for longer than 3 months) is more often seen after mild traumatic brain injury (mTBI) than after moderate to severe TBI [4–6]. It is estimated that 19 to 28% of patients with mTBI will still have headache at 1 year after the trauma [7, 8].

Pre-existing migraine is a risk factor for developing persistent PTH [9]. Yet, the effect of TBI on headache features, associated symptoms, triggers, and comorbidities among patients with migraine is not well known. Likewise, a history of head or neck injury is also a risk factor for migraine chronification [10]. Moreover, headache is one of the most common post-concussive symptoms, occurring up to 80%% of patients [11], and more than 60% have headache features that are consistent with those seen in migraine or probable migraine [12].

This study aimed to investigate the effect of mTBI history in migraine patients enrolled in the American Registry for Migraine Research (ARMR) [13] by examining differences in migraine-related symptoms, clinical attributes, and associated disability among patients diagnosed with migraine with and without a history of mTBI.

## Methods

### Design and setting

The ARMR, established by the American Migraine Foundation, is a multicenter, longitudinal patient registry that collected participants’ clinical data, imaging, and biospecimens. Patients were recruited and enrolled from ARMR sites, which are specialty headache clinics in the United States [13]. Institutional Review Board approvals were obtained from each of the enrolling sites, and all participants completed an informed consent process. Headache specialists assigned one or more ICHD-3 diagnoses to each patient, which were entered into ARMR by a clinician or research staff member. According to ARMR inclusion criteria, participants had to be at least 18 years of age, able and willing to provide informed consent, and able to access a computer or other electronic device with an internet connection. No a priori statistical power calculation was conducted. The sample size of the data for this research analysis was based on the available data, collected via questionnaires completed by patients at the time they enrolled into ARMR between February 1, 2016 to March 6, 2020.

### Study population /inclusion and exclusion criteria

**Fig. 1:**
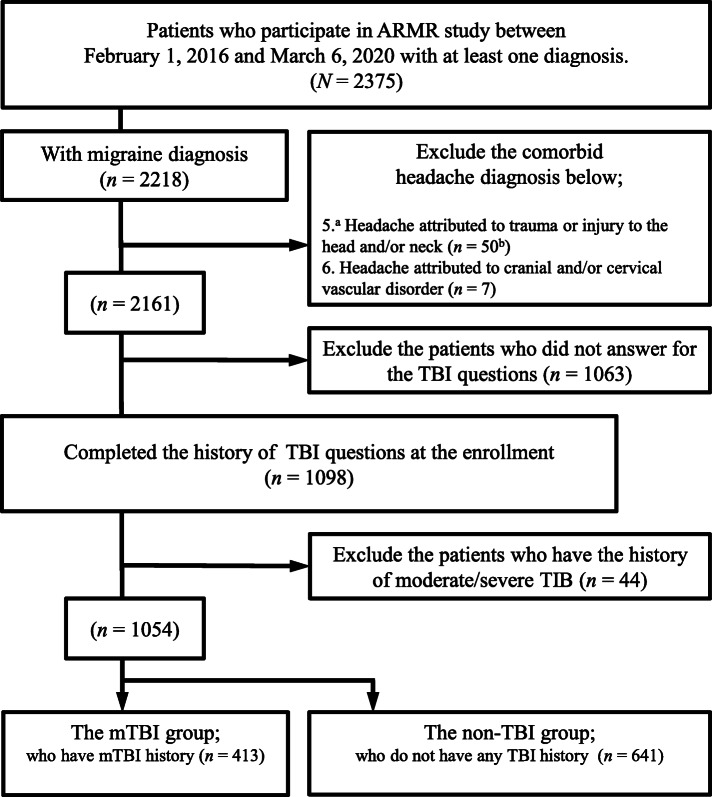
Flow-chart summarizing inclusion and exclusion of patients ^a^The first numbers indicate the ICHD-3 diagnosis code. ^b^The last numbers indicate the number of patients excluded because of having that headache or facial pain type. ARMR: American Registry for Migraine Research, TBI: traumatic brain injury, mTBI: mild traumatic brain injury.

Of 2218 patients who participated in the ARMR study, and were assigned an ICHD migraine diagnosis between February 2016 and March 2020, 57 (2.6%) individuals were excluded due to having one or more headache diagnoses in addition to migraine: headache attributed to trauma or injury to the head and/or neck (i.e. PTH) (*n* = 50) or headache attributed to cranial and/or cervical vascular disorder (*n* = 7). Among the remaining 2161 patients, 1098 (50.8%) completed the questions about history of TBI. The question was “Have you ever had a head injury? A head injury may be a hit to the head that caused headaches, dazed feeling, mental fogginess, lightheadedness, blurred vision, dizziness or vomiting.” and participants could answer by two alternatives Yes/ No. Forty-four patients with moderate/severe TBI were excluded. In accordance with ICHD-3 criteria, moderate/severe TBI was defined as TBI with loss of consciousness exceeding 30 min or post-traumatic amnesia exceeding 24 h. Twenty-five excluded patients had loss of consciousness for more than 30 min and 25 had post-traumatic amnesia for more than 24 h (6 patients had both symptoms). The patients were divided into 2 subgroups: the mTBI group (*n* = 413) who had a history of mTBI, and the non-TBI group (*n* = 641) who did not have a history of any TBI (Fig. 1).

### Data extraction

Data extracted from the ARMR database included the clinician’s assigned ICHD-3 headache diagnosis, and patient-reported age, gender, race, education level, marital status, employment status, household income, history of any type of abuse, and history of physical abuse. TBI characteristics included: severity of TBI, worsening headache or new headache after the TBI, duration of new/worsening headache within 7 days after TBI, and time elapsed between most recent TBI and enrollment. Headache-related symptoms included: headache triggers, average monthly headache frequency over the last 3 months, duration of headache history (years since first headache), headache intensity using a Numeric Rating Scale (NRS) [14, 15], how often the headache-related symptoms (nausea, vomiting, dizziness, vertigo, sensitivity to light, sensitivity to noise, and sensitivity to smell, difficulty finding words) were occurred during headache attack, subjective cognitive impairment during migraine attacks (Mig-SCog) score [16], and Allodynia Symptom Checklist-12 (ASC-12) scores [17]. Disability data included: Migraine Disability Assessment Scale (MIDAS) score [18], Work Productivity and Activity Impairment due to headache (WPAI) scores [19], and Patient-Reported Outcomes Measurement Information System- Pain Interference (PROMIS-PI) score [20]. Coexisting headache diagnoses extracted include: Migraine with aura (MwA) and Medication-Overuse Headache (MOH). Psychiatric comorbidity data extracted included: Patient Health Questionnaire-2 (PHQ-2) score [21], General Anxiety Disorder-7 (GAD-7) score [22], and self-reported history of post-traumatic stress disorder (PTSD).

NRS is a commonly used scale in which a person rates their pain from 0 (no pain) to 10 (worst pain) [14, 15]. The Mig-SCog score measures subjective cognitive impairment during migraine attacks [16]. The ASC-12 assesses cutaneous allodynia symptoms during headache attacks [17]. The MIDAS questionnaire quantifies headache-related disability over the prior 3-month period [18]. WPAI evaluates disability and impact on work productivity due to a specific health problem amongst those who are employed [19]. Based on answers about the past 7 days, four different scores are calculated (WPAI-%Absenteeism; percent work time missed due to headache, WPAI-%Presenteeism; percent impairment while working due to headache, WPAI-%Total Work Productivity Impairment; percent overall work impairment due to headache, and WPAI-%Total Activity Impairment; percent activity impairment due to headache). The PROMIS-PI Adult Short Form 6b measures pain interference on functioning and quality of life over the past 7 days. PROMIS-PI raw scores can be converted to standardized T scores by using conversion tables available at http://www.nihpromis.org/, so that the scores can be compared with the score of the United States general population and used as a normal distribution [20]. The PHQ-2 score is a valid brief tool for detecting depressive disorders over the last 2 weeks [21]. GAD-7 assesses the severity of general anxiety disorder symptoms [22].

### Statistical analysis

Descriptive statistics are presented as mean ± standard deviation, median (interquartile range: [IQR]), or number (percentage). Patient characteristics were compared using the chi-square test for categorical variables. Fisher’s exact tests were used for comparing headache triggers, since some categories included a count of fewer than 5, which is not suitable for chi-square-test comparison. Welch’s t-test was performed for PROMIS-PI-T scores. The Mann-Whitney test was used for the other continuous variables because a series of Shapiro-Wilk-tests revealed that all the continuous variables in this study were not normally distributed. In the analysis, missing data were handled using pairwise deletion.

To assess differences in gender between mTBI group and non-TBI group, we excluded transgender patients, since the number of transgender patients was small (*n* = 1 for mTBI group and 2 for non-TBI group). Similarly, because the numbers of patients identifying as race categories other than white were also small, we thus combined all patients who did not identify as white into one category for purposes of analysis. To assess the difference between two groups in age at onset of headache, the Log-rank test was performed. To describe the characteristics of migraine patients with mTBI regardless of having Chronic migraine (CM), logistic regression models adjusted for age, gender, and CM were performed. *P*-values < 0.05 on likelihood ratio tests were considered significant. Data were analyzed using Graph Pad PRISM for Windows (version 5.04, Graph Pad Software, Inc., CA, USA), JMP for Windows (version 11.2.0 m SAS Institute Inc., NC, USA) and R statistical computing program (version 3.2.2, https://www.r-project.org/).

## Results

### Sociodemographics

Sociodemographics and diagnoses are shown in Table 1. The mean age was 46 ± 13 years. Participants were predominantly female (*n* = 929; 88.1%), white (*n* = 975; 94.1%), with a college or graduate degree (*n* = 955; 94.2%), employed full- or part-time (*n* = 620; 60.3%), and married or living with domestic partner (*n* = 703; 67.8%). The most common household income category was between $50,000 and $99,999 (*n* = 306; 31.6%). The proportion of patients who reported being married or in a domestic partnership in the mTBI group was significantly lower than that of the non-TBI groups (63.4% [255/402] vs. 70.6% [449/636], *P* = 0.016). There was no significant difference in age, gender, race, educational level, employment status, or household income between the two groups. These results were consistent with those of the logistic regression model adjusted for age, gender, and CM.

**Table 1:** Subject sociodemographics and headache diagnoses

	Total		mTBI group		non-TBI group		*P*-value^a^	Gender, age, CM controlled *P*-value^f^
Number	1054	(%)	413	(%)	641	(%)		
Age								
Mean ± SD	46	± 13	46	± 13	45	± 13	0.537^c^	–
Median (IQR)	46	(35–56)	46	(35–57)	46	(35–55)		
Gender								
Female	929	(88.1%)	356	(86.2%)	573	(89.4%)	0.107^b,d^	–
Male	122	(11.6%)	56	(13.6%)	66	(10.3%)		
Transgender	3	(0.3%)	1	(0.2%)	2	(0.3%)		
Race								
White	975	(94.1%)	384	(95.0%)	591	(93.5%)	0.069^b,e^	0.074
Black/African	23	(2.2%)	6	(1.5%)	17	(2.7%)		
Asian	15	(1.4%)	4	(1.0%)	11	(1.7%)		
American Indian/ Alaska	18	(1.7%)	8	(2.0%)	10	(1.6%)		
Hawaiian/Other Pacific	5	(0.5%)	2	(0.5%)	3	(0.5%)		
Unknown/ Missing	18	–	9	–	9	–		
Education level								
High school or less	59	(5.8%)	27	(6.9%)	32	(5.1%)	0.242^b^	0.292
College or degree level	955	(94.2%)	364	(93.1%)	591	(94.9%)		
Missing	40	–	22	–	18	–		
Marital status								
Married/Domestic partnership	703	(67.8%)	255	(63.4%)	449	(70.6%)	**0.016**^**b**^	**0.007**
Never married/Separated/Widowed	334	(32.2%)	147	(36.6%)	187	(29.4%)		
Missing	16	–	11	–	5	–		
Employment								
Full-time/part time	620	(60.3%)	235	(58.8%)	385	(61.2%)	0.432^b^	0.664
not employee	409	(39.7%)	165	(41.3%)	244	(38.8%)		
Missing	25	–	13	–	12	–		
Household income								
Under $50,000	198	(20.4%)	85	(22.5%)	113	(19.1%)	0.385^b^	0.421
$50,000 to $99,999	306	(31.6%)	117	(31.0%)	189	(32.0%)		
$100,000 to $149,999	224	(23.1%)	89	(23.5%)	135	(22.8%)		
$150,000 to $199,999	113	(11.7%)	46	(12.2%)	67	(11.3%)		
$200,000 or more	128	(13.2%)	41	(110.8%)	87	(14.7%)		
Missing	85	–	35	–	50	–		

^a^Bolded text indicates significant difference. ^b^Chi-square test. ^c^Mann-Whitney test (*p*-value of Shapiro-Wilk test was < 0.05). ^d^Analysis included only female and male, due to small number of transgender participants. ^e^Since there were a relatively small number of non-white participants, these groups were combined for purposes of analysis using chi-square-test. ^f^Logistic regression model was performed with controlling for age, gender, and chronic migraine

*CM* Chronic Migraine, *IQR* Interquartile range, *mTBI* mild traumatic brain injury, *SD* significant difference

### Characteristics of TBI among mTBI group

Of 413 patients in mTBI group, 268 (64.8%) of patients had headaches prior to their head injury, while 139 (33.7%) of them did not have headaches prior to their head injury (missing data = 6). The latest TBI event occurred an average of 18.3 ± 16.0 (median [IQR]; 14.4 [4.7–28.5]) years before enrollment into ARMR. Despite not having a clinician-assigned ICHD diagnosis of PTH, one hundred and forty-five patients reported that they had new/worsening headache within 7 days of their TBI (Table 2).

**Table 2:** Time course of head injury and its characteristics among TBI group

	mTBI group	
Number	413	
Having headaches before head injury		
Yes	268	(64.8%)
No	139	(33.7%)
Missing	6	–
Duration between head injury and baseline enrollment (years)		
Mean ± SD	18.3 ± 16.0	
Median (IQR)	14.4 (4.7–28.5)	
Either new headache or worsening headache after the head injury		
Yes	145	
No	189	
I don’t know/Missing	79	
Either new headache or worsening headache within 7 days after the head injury		
Yes	92	
No	27	
I don’t know/Missing	294	

*IQR* interquartile range, *mTBI* mild traumatic brain injury, *SD* significant difference

### Headache phenotype and associated symptoms

Amongst our study population, 27.3% (288/1054) had MwA, 69.2% (729/1054) had CM, and 6.3% (66/1054) had MOH as one of the registered multiple diagnoses. Among the total sample, 47.7% (503/1054) of patients had nausea, 7.0% (74/1054) had vomiting, 41.0% (432/1054) had headache with non-vertigo dizziness, 18.7% (197/1053) had vertigo-type dizziness, 74.8% (788/1053) had sensitivity to light, 71.1% (748/1053) had sensitivity to noise, 48.9% (513/1052) had sensitivity to smell, and 36.8% (382/1050) had difficulty finding words in half or more of their headache attacks.

The proportion of patients with a CM diagnosis in the mTBI group was significantly higher than in the non-TBI group (74.3% [307/413] vs. 65.8% [422/641]; *p* = 0.004). These were no significant differences between two groups in the proportion of having MwA (27.8% [115/413] vs. 27.0% [173/641], *P* = 0.761) and MOH (6.8% [28/413] vs. 5.9% [38/641], *P* = 0.578). Patients in the mTBI group were more likely to have non-vertigo dizziness (*P* = 0.006), vertigo (*P* = 0.010), and difficulty finding words (*P* < 0.001) compared to the patients in non-TBI group. Patients in the mTBI group had significantly greater allodynia scores (median [IQR] 5 [1–9] vs. 4 [1–7], *P* < 0.001] compared to the patients in the non-TBI group. The patients in the mTBI group had significantly higher Mig-SCog scores (8.9 ± 4.9 vs. 7.5 ± 4.7, *P* < 0.001) compared to the non-TBI group. There were no significant differences between two groups for nausea (*P* = 0.159), vomiting (*P* = 0.445), sensitivity to light (*P* = 0.096), sensitivity to noise (*P* = 0.433), and sensitivity to smell (*P* = 0.415). After adjusting for age, gender, and CM, significant differences were consistent for dizziness (*P* = 0.003), vertigo (*P* = 0.009), allodynia score (*P* < 0.001), difficulty finding words (*P* < 0.001), and Mig-Scog score (*P* < 0.001) (Table 3).

**Table 3:** Headache symptoms

	Total		mTBI group		non-TBI group		*P*-value^a^	Gender, age, CM controlled *P*-value^d^
Number	1054	(%)	413	(%)	641	(%)		
Diagnoses								
MwA	288	(27.3%)	115	(27.8%)	173	(27.0%)	0.761^b^	0.588
CM	729	(69.2%)	307	(74.3%)	422	(65.8%)	**0.004**^**b**^	–
MOH	66	(6.3%)	28	(6.8%)	38	(5.9%)	0.578^b^	0.803
Nausea								
Always	152	(14.4%)	65	(15.7%)	87	(13.6%)	0.159^b^	0.081
Half the time or more	351	(33.3%)	142	(34.4%)	209	(32.6%)		
Less than half the time	269	(25.5%)	113	(27.4%)	156	(24.3%)		
Rarely	237	(22.5%)	79	(19.1%)	158	(24.6%)		
Never	45	(4.3%)	14	(3.4%)	31	(4.8%)		
Missing	0	–	0	–	0	–		
Vomiting								
Always	8	(0.8%)	4	(1.0%)	4	(0.6%)	0.445^b^	0.272
Half the time or more	66	(6.3%)	29	(7.0%)	37	(5.8%)		
Less than half the time	167	(15.8%)	74	(17.9%)	93	(14.5%)		
Rarely	437	(41.5%)	166	(40.2%)	271	(42.3%)		
Never	376	(35.7%)	140	(33.9%)	236	(36.8%)		
Missing	0	–	0	–	0	–		
Dizziness: Nonvertigo (The feeling of unsteadiness, lightheadedness, motion sickness)								
Always	136	(12.9%)	63	(15.3%)	73	(11.4%)	**0.006**^**b**^	**0.003**
Half the time or more	296	(28.1%)	130	(31.5%)	166	(25.9%)		
Less than half the time	264	(25.0%)	104	(25.2%)	160	(25.0%)		
Rarely	237	(22.5%)	83	(20.1%)	154	(24.0%)		
Never	121	(11.5%)	33	(8.0%)	88	(13.7%)		
Missing	0	–	0	–	0	–		
Vertigo/Dizziness (The feeling that you or your environment is moving or spinning; an illusion of movement)								
Always	47	(4.5%)	22	(5.3%)	25	(3.9%)	**0.010**^**b**^	**0.009**
Half the time or more	150	(14.2%)	73	(17.7%)	77	(12.0%)		
Less than half the time	205	(19.5%)	89	(21.5%)	116	(18.1%)		
Rarely	348	(33.0%)	127	(30.8%)	221	(34.5%)		
Never	303	(28.8%)	102	(24.7%)	201	(31.4%)		
Missing	1	–	0	–	1	–		
Sensitivity to light								
Always	480	(45.6%)	204	(49.4%)	276	(43.1%)	0.096^b^	0.090
Half the time or more	308	(29.2%)	123	(29.8%)	185	(28.9%)		
Less than half the time	130	(12.3%)	45	(10.9%)	85	(13.3%)		
Rarely	107	(10.2%)	32	(7.7%)	75	(11.7%)		
Never	28	(2.7%)	9	(2.2%)	19	(3.0%)		
Missing	1	–	0	–	1	–		
Sensitivity to noise								
Always	444	(42.2%)	186	(45.1%)	258	(40.3%)	0.433^b^	0.402
Half the time or more	304	(28.9%)	116	(28.2%)	188	(29.4%)		
Less than half the time	147	(14.0%)	53	(12.9%)	94	(14.7%)		
Rarely	116	(11.0%)	45	(10.9%)	71	(11.1%)		
Never	41	(3.9%)	12	(2.9%)	29	(4.5%)		
Missing	2	–	1	–	1	–		
Sensitivity to smell								
Always	281	(26.8%)	123	(29.9%)	158	(24.8%)	0.415^b^	0.350
Half the time or more	232	(22.1%)	88	(21.4%)	144	(22.6%)		
Less than half the time	156	14.9%)	61	(14.8%)	95	(14.9%)		
Rarely	191	(18.2%)	73	(17.7%)	118	(18.5%)		
Never	190	(18.1%)	67	(16.3%)	123	(19.3%)		
Missing	4	–	1	–	3	–		
Allodynia Score (Allodynia Symptom Checklist)								
Mean (SD)	5.1	±4.8	5.8	±5.1	4.6	±4.5	**< 0.001**^**c**^	**< 0.001**
Median (IQR)	4	(1–8)	5	(1–9)	4	(1–7)		
Missing	120	–	42	–	78	–		
Difficulty finding words, expressing yourself verbally or in writing, or fully understanding what others are saying								
Always	125	(12.0%)	61	(15.1%)	64	(10.1%)	**< 0.001**^**b**^	**< 0.001**
Half the time or more	257	(24.8%)	113	(27.9%)	144	(22.7%)		
Less than half the time	216	(20.8%)	86	(21.2%)	130	(20.5%)		
Rarely	210	(20.2%)	82	(20.2%)	128	(20.2%)		
Never	230	(22.2%)	63	(15.6%)	167	(26.4%)		
Missing	16	–	8	–	8	–		
Subjective Cognitive Impairment (Mig-SCog)								
Mean (SD)	8.1	±4.8	8.9	± 4.9	7.5	± 4.7	**< 0.001**^**c**^	**< 0.001**
Median (IQR)	8	(4–12)	9	(5–13)	7	(4–11)		
Missing	35		20		15			

^a^Bolded text indicates significant difference. ^b^Chi-square test. ^c^Mann-Whitney test (*p*-value of Shapiro-Wilk test was < 0.05). ^d^Logistic regression model was performed with controlling for age, gender, and chronic migraine. *CM* Chronic migraine, *MwA* migraine with aura, *MOH* medication-overuse headache, *mTBI* mild traumatic brain injury, *SD* significant difference, *IQR* interquartile range, *Mig-SCog* Subjective cognitive impairment during migraine attacks

### Headache triggers

Headache triggers are summarized in Table 4. About one third of all migraine patients had headaches that were always triggered by lack of sleep (35.3% [353/1000]), stress (30.8% [308/1000]), and weather change (29.8% [298/1000]). Patients in the mTBI group were more likely to describe that their headaches were always triggered by lack of sleep (39.4% [155/393] vs. 30.9% [198/607], *P* = 0.032), bright lights (21.4% [84/393] vs. 15.4% [99/607], *P* = 0.045), and reading (6.6% [26/393] vs. 2.8% [18/607], *P* = 0.007) compared to non-TBI patients in the initial analysis. After adjusting for age, gender, and CM diagnosis, only lack of sleep (*P* = 0.018) and reading (*P* = 0.016) maintained a significant difference between the mTBI and non-TBI groups. There were no significant differences between the two groups in other triggers.

**Table 4:** Headache triggers

	Total		mTBI group		non-TBI group		*P*-value^a^	Gender, age, CM controlled *P*-value^c^
Number	1054	(%)	413	(%)	641	(%)		
Lack of sleep	353	(35.3%)	155	(39.4%)	198	(30.9%)	**0.032**^**b**^	**0.018**
Stress	308	(30.8%)	127	(32.3%)	181	(28.2%)	0.441^b^	0.314
Weather change	298	(29.8%)	123	(31.3%)	175	(27.3%)	0.436^b^	0.483
Bright lights	183	(18.3%)	84	(21.4%)	99	(15.4%)	**0.045**^**b**^	0.067
Menstruation	150	(15%)	51	(13.0%)	99	(15.4%)	0.174^b^	0.208
Fatigue	148	(14.8%)	60	(15.3%)	88	(13.7%)	0.738^b^	0.811
Huger	143	(14.3%)	58	(14.8%)	85	(13.3%)	0.782^b^	0.862
Odors	135	(13.5%)	47	(12.0%)	88	(13.7%)	0.258^b^	0.208
Releif from stress	117	(11.7%)	49	(12.5%)	68	(10.6%)	0.547^b^	0.455
Alcohol	113	(11.3%)	43	(10.9%)	70	(10.9%)	0.838^b^	0.702
Over heated	91	(9.1%)	33	(8.4%)	58	(9.0%)	0.575^b^	0.493
Food	82	(8.2%)	30	(7.6%)	52	(8.1%)	0.638^b^	0.541
Loud sounds	82	(8.2%)	34	(8.7%)	48	(7.5%)	0.724^b^	0.912
Smoke	80	(8.0%)	29	(7.4%)	51	(8.0%)	0.634^b^	0.442
Screen time	74	(7.4%)	35	(8.9%)	39	(6.1%)	0.173^b^	0.198
Altitude change	72	(7.2%)	28	(7.1%)	44	(6.9%)	1.000^b^	0.770
Physical exertion	71	(7.1%)	25	(6.4%)	46	(7.2%)	0.529^b^	0.348
Too much sleep	44	(4.4%)	22	(5.6%)	22	(3.4%)	0.156^b^	0.156
Reading	44	(4.4%)	26	(6.6%)	18	(2.8%)	**0.007**^**b**^	**0.016**
Visual patterns	41	(4.1%)	19	(4.8%)	22	(3.4%)	0.415^b^	0.314
Caffeine	34	(3.4%)	11	(2.8%)	23	(3.6%)	0.477^b^	0.435
Medicine	30	(3.0%)	11	(2.8%)	19	(3.1%)	0.851^b^	0.700
Coughing/sneezing	30	(3.0%)	17	(4.3%)	13	(2.0%)	0.058^b^	0.090
Sex/orgasm	12	(1.2%)	6	(1.5%)	6	(0.9%)	0.555^b^	0.490
Nonalcoholic beverage	11	(1.1%)	4	(1.0%)	7	(1.1%)	1.000^b^	0.710
I don’t know	191	(19.1%)	64	(16.3%)	127	(19.8%)	0.071^b^	0.060
Missing	54	–	20	–	34	–		

^a^Bolded text indicates significant difference. ^b^Fisher’s exact test. ^c^Logistic regression model was performed, controlling for age, gender, and chronic migraine. *CM* Chronic Migraine, *mTBI* mild traumatic brain injury

### Headache features and disability

Headache features and disability measures are shown in Table 5, including: headache frequency, duration of headache history, age at onset of headache, NRS and scores on MIDAS, WPAI, and PROMIS-PI-T. Among the total population, median headache days per month was 12.0 (IQR; 6.0–25.0), median duration of headache history was 19.3 (IQR; 7.4–31.8) years, median age at onset of headache was 20 (IQR; 13–34), median NRS was 6 (IQR; 5–7), and the mean PROMIS-PI-T score of the total patient sample was 63.2 ± 7.0. MIDAS scores of the TBI group were significantly higher than those of the non-TBI group (median [IQR]; 42 [18–85] vs. median 34.5 [15–72], *P* = 0.010). The patients in the mTBI group had significantly higher WPAI-%Absenteeism (12.8 ± 22.2 vs. 8.7 ± 17.2, *P* = 0.012) compared to the non-TBI group. After adjusting for age, gender, and CM, significant differences remain between groups with and without history of mTBI in MIDAS scores (*P* = 0.035), and WPAI-%Absenteeism (*P* = 0.031).

**Table 5:** Headache Characteristics, Disability, Anxiety and Depression

	Total	mTBI group	non-TBI group	*P*-value^a^	Gender, age, CM controlled *P*-value^e^
Number	1054	413	641		
Headache days per month					
Mean (SD)	14.6 ± 10.0	15.0 ± 10.1	14.3 ± 9.9	0.283^b^	0.722
Median (IQR)	12.0 (6.0–25.0)	13.0 (6.0–25.3)	11.2 (5.7–23.7)		
Missing	108	41	67		
Duration of headache history (years)					
Mean (SD)	21.4 ± 15.8	22.7 ± 16.7	20.5 ± 15.1	0.132^b^	0.149
Median (IQR)	19.3 (7.4–31.8)	20.1 (8.2–34.4)	18.7 (7.2–30.7)		
Missing	248	95	153		
Age at onset of headache					
Mean (SD)	24.4 ± 14.4	23.5 ± 14.2	24.9 ± 14.5	0.116^d^	0.051
Median (IQR)	20 (13–34)	19 (13–34)	21 (13–34)		
Ratio		0.90 (0.03–1.78)			
Hazard Ratio		1.12 (0.97–1.30)			
Missing	216	84	135		
Pain NRS					
Mean (SD)	6.0 ± 1.7	6.0 ± 1.7	6.0 ± 1.7	0.857^b^	0.963
Median (IQR)	6 (5–7)	6 (5–7)	6 (5–7)		
Missing	103	40	63		
MIDAS total score					
Mean (SD)	53.3 ± 49.9	58.6 ± 52.6	50.0 ± 47.8	**0.010**^**b**^	**0.034**
Median (IQR)	38 (15–80)	42 (18–85)	34.5 (15–72)		
Missing	118	47	71		
WPAI-% Absenteeism (work time missed due to migraine)					
Mean (SD)	10.3 ± 19.4	12.8 ± 22.2	8.7 ± 17.2	**0.012**^**b**^	**0.031**
Missing	495	195	300		
WPAI-% Presenteeism (impairment while working due to migraine)					
Mean (SD)	37.7 ± 25.4	39.6 ± 25.5	36.5 ± 25.2	0.385^b^	0.312
Missing	495	195	300		
WPAI-Total work productivity impairment					
Mean (SD)	37.8 ± 26.4	40.0 ± 27.1	36.4 ± 26.3	0.140^b^	0.229
Missing	495	195	300		
WPAI-% activity impairment due to migraine					
Mean (SD)	41.9 ± 26.7	44.4 ± 26.0	40.4 ± 27.1	0.148^b^	0.200
Missing	521	299	222		
PROMIS-PI-Tscore					
Mean (SD)	63.2 ± 7.0	63.6 ± 7.1	62.9 ± 7.0	0.101^c^	0.241
Missing	109	43	66		

^a^Bolded text indicates significant difference. ^b^Mann-Whitney test (*p*-value of Shapiro-Wilk test was < 0.05). ^c^Welch’s t-test. ^d^Log-rank test. ^e^Logistic regression model was performed, controlling for age, gender, and chronic migraine

*CM* Chronic Migraine, *IQR* interquartile range, *MIDAS* Migraine Disability Assessment Scale, *mTBI* mild traumatic brain injury, *NRS* Numeric Rating Scale, PROMIS- P I: Patient-Reported Outcomes Measurement Information System-Pain Interference, *SD* significant difference, *WPAI* Work Productivity and Activity Impairment

There were no significant differences between two groups in WPAI-%Presenteeism (39.6 ± 25.5 vs. 36.5 ± 25.2, *P* = 0.385), WPAI-%Total Work Productivity Impairment (40.0 ± 27.1 vs. 36.4 ± 26.3, *P* = 0.140), WPAI-%Activity Impairment due to migraine (44.4 ± 26.0 vs. 40.4 ± 27.1, *P* = 0.148), or PROMIS-PI-T scores (63.6 ± 7.1 vs. 62.9 ± 7.0, *P* = 0.101). Further, there was no significant difference between groups with and without history of mTBI in headache frequency (median [IQR]; 13.0 days [6.0–25.3] vs. 11.2 [5.7–23.7], *P* = 0.283], duration of headache history (median [IQR]; 20.1 years [8.2–34.4] vs. 18.7 [7.2–30.7], *P* = 0.132), age at onset of headache (23.5 ± 14.2 vs. 24.9 ± 14.5, Hazard Ratio (developing headache per unit period) [Confidence Interval (CI)]; 1.12 [0.97–1.30], *P* = 0.116), and NRS (median [IQR]; 6 [5–7] vs. 6 [IQR; 5–7], *P* = 0.857).

### History of abuse and psychiatric disorders

In total, 36.8% (308/836, missing data = 218) of migraine patients self-reported a history of abuse in their lifetime, in particular physical abuse history (18.4% [154/836], missing data = 218). PTSD was seen in 26.2% (122/465, missing data = 589) of the total population. A higher proportion of patients in the mTBI group self-reported a history of any type of abuse (47.0% [162/345] vs. 29.7% [146/491], *P* < 0.001), including the physical abuse subtype (24.3% [84/345] vs. 14.3% [70/491], *P* < 0.001). The patients in the mTBI group also had significantly higher PHQ-2 (*P* = 0.034) scores and a greater proportion had mild to severe GAD-7 grades (*P* = 0.003). A greater proportion of patients with a history of mTBI self-reported a history of PTSD (33.3% [68/204] vs. 20.7% [54/261], *P* = 0.002) compared to the non-TBI group. After adjusting for age, gender, and CM, significant differences between groups remain for history of abuse (*P* < 0.001), history of physical abuse (*P* < 0.001), PHQ-2 scores (*P* = 0.012), GAD-7 scores (*P* = 0.003), and history of PTSD (*P* = 0.002) (Table 6).

**Table 6:** Past history of abuse and psychiatric disorders

	Total	mTBI group	non-TBI group	*P*-value^a^	Gender, age, CM controlled *P*-value^c^
Number	1054	413	641		
Any history of abuse					
Yes	308 (36.8%)	162 (47.0%)	146 (29.7%)	**< 0.001**^**b**^	**< 0.001**
No	528 (63.2%)	183 (53.0%)	345 (70.3%)		
Missing	218	68	150		
History of physical abuse					
Yes	154 (18.4%)	84 (24.3%)	70 (14.3%)	**< 0.001**^**b**^	**< 0.001**
No	682 (81.6%)	261 (75.7%)	421 (85.7%)		
Missing	218	68	150		
Depression (PHQ-2 Score)					
Mean (SD)	1.4 ± 1.7	1.6 ± 1.8	1.3 ± 1.6	**0.034**^**d**^	**0.012**
Median (IQR)	1 (0–2)	1 (0–2)	1 (0–2)		
Missing	115	43	72		
Anxiety (GAD-7 grade)					
Minimal	573 (55.3%)	201 (49.5%)	372 (59.0%)	**0.003**^**b**^	**0.003**
Mild-Severe	463 (44.7%)	205 (50.5%)	258 (41.0%)		
Missing	18	7	11		
PTSD					
Yes	122 (26.2%)	68 (33.3%)	54 (20.7%)	**0.002**^**b**^	**0.002**
No	343 (73.8%)	136 (66.7%)	207 (79.3%)		
Missing	589	209	380		

^a^Bolded text indicates significant difference. ^b^Chi-square test. ^c^Logistic regression model was performed, controlling for age, gender, and chronic migraine. ^d^Mann-Whitney test. *CM* Chronic Migraine, *GAD-7* General Anxiety Disorder-7, *IQR* interquartile range, *mTBI* mild traumatic brain injury, *PHQ-2* Patient Health Questionnaire-2, *PTSD* Post-traumatic stress disorder, *SD* significant difference

## Discussion

### Migraine severity and related disability were greater in those with a history of mTBI

Within our study population, 38% of patients with migraine reported a history of mTBI, which occurred a median of 14.4 (IQR; 4.7–28.5) years before enrollment into ARMR. This proportion is higher than expected in the general population (19.1% lifetime prevalence of mTBI) [23]. Further, when compared with non-TBI patients, a significantly greater proportion of patients with a history of mTBI had CM. While, TBI has been reported as an independent risk factor for CM [24, 25], the occurrence of chronic daily headache is not necessarily related to the proximity of the TBI [4].

The patients in the mTBI group had significantly greater headache-related disability (higher MIDAS scores) over the prior 3 months and more work productivity impairment due to headache in the past 7 days (WPAI-%Absenteeism), compared to those without a history of mTBI. This underscores the likelihood that a history of mTBI is associated with a more severe clinical phenotype of migraine that may persist for many years after the TBI event, regardless of having a CM diagnosis.

### Remote effect of mTBI on migraine

While a history of mTBI did not appear to significantly affect the age of migraine onset, this study found that even a long-ago history of TBI (median 14.4 years) might have a durable effect on migraine, including severity, disability, and associated features such as vertigo, cognitive dysfunction, allodynia, and psychological comorbidities. These remote effects are compatible with previous reports. A prior study following veterans with deployment-related TBI for 4–11 years after injury reported more frequent and severe headaches compared to the age-, sex-, and race-matched veterans without TBI [26]. Moreover, the vast majority of these veterans (89%) experienced PTH with a phenotype of migraine. The authors concluded that there was no improvement in headache prevalence or frequency over time, suggesting that the process related to headache causation initiated by the TBI either remained active or produced permanent changes in brain function allowing the headaches to continue for a protracted time. This is further supported by emerging data in blood-based biomarkers, showing persistent elevation of neurofilament light chain, up to 22 years after an initial mTBI [27]. Such findings raise the possibility of persistent neuroinflammation and pathological cascades long after mTBI. In addition to structural and biochemical changes in the brain, TBI-induced psychiatric disorders such as depression, anxiety, and PTSD may also continuously increase the severity of migraine [28–30]. Overall, these results suggest that, while a history of mTBI may not affect the onset of migraine, but that there are measurable remote effects chronification and severity.

### Headache-associated symptoms differ in those with a history of mTBI compared to those without

Consistent with prior studies [31], our study found that the patients in the mTBI group reported having significantly more vestibular dysfunction, including dizziness and vertigo after adjusting for age, gender, and CM. Another study reported that half of TBI patients have vestibular complaints at 5 years after their TBI [32], and some authors have noted an association between vestibular symptoms and headache in blast-exposed soldiers, hypothesizing this to be suggestive of a “migraine-like” headache phenotype. The authors speculated that this association might suggest a potential migraine biology underlying the vestibular and balance dysfunction following concussion [33].

Allodynia has been reported in an estimated 35% of military members with PTH and 42–46% of civilians with PTH [34–36], leading to the hypothesis that mTBI may increase the risk for allodynia [37, 38] and CM through altered descending modulation of trigeminal sensory processing [39]. The estimated frequency of allodynia among people with migraine is perhaps more variable (15.1% to 69.7%) [40], largely depending on chronicity, with a higher prevalence in CM than in episodic migraine patients [41, 42]. In our study, patients in the mTBI group were more likely to report significant headache-associated allodynia (higher ASC-12 scores), compared to those in the non-TBI group. Interestingly, this was true even *after* adjusting for CM, suggesting a potential independent impact of TBI history on the manifestation of headache-associated allodynia. As with vertigo and dizziness, the presence of allodynia in a patient presenting with a CM phenotype should elicit queries of a TBI history.

It is well known that migraine attacks are associated with poorer cognitive performance than headache-free periods, consistent with cognitive difficulties subjectively reported during attacks. Most clinic-based studies report worse cognitive performance in people with migraine compared to those without [43]. In particular, verbal skills (auditory comprehension, reading, aphasia screening, verbal reasoning, vocabulary, and phoneme detection) of people with migraine were reported to be mildly impaired [44]. It is also known that cognitive impairment is a common and disabling consequence of TBI [45], and mTBI has been demonstrated to be an independent risk factor for dementia [46, 47]. Prolonged cognitive impairment is also described in patients with PTH, particularly in those with a history of headache prior to the TBI event [48].

Our findings further support these observations, with higher Mig-SCog scores and greater difficulty finding words reported by the mTBI group, compared to the patients in the non-TBI group (Tables 3 and 5). These results suggest that people with migraine and a history of mTBI, regardless of whether they have CM, should be assessed for persistent cognitive dysfunction, and support ongoing investigations into the extent to which people with migraine who have a history of mTBI have an elevated risk of later-life cognitive impairment.

### Triggers of headache in mTBI group

Lack of sleep and reading were the most notable headache triggers reported by the mTBI group in our study, despite the fact that the mTBI event occurred a median of 14.4 years prior to study participation. In general, sleep deprivation is a common, well-recognized trigger of migraine. One of the largest retrospective surveys reported sleep disturbance as a trigger in 49.8% of migraineurs [49]. On the other hand, reading as a trigger (or precipitating) factor of migraine has been reported in 4–18%. The proportion of migraine patients with migraine attacks induced by reading was not significantly different from that of tension-type headaches induced by reading, in the previous studies [50, 51]. Reading may trigger headache after TBI via oculomotor dysfunction [52], verbal dysfunction, or cognitive dysfunction [45] after TBI. The TBI was reported to result in long-lasting oculomotor dysfunction [53], as well as verbal or cognitive dysfunction [54].

### Physical abuse and psychological comorbidities

Anxiety and depression are widely recognized comorbidities in migraine, and serve as risk factors for CM [55, 56]. Further, mTBI can independently provoke or aggravate anxiety and depression [57, 58]. Finally, a history of trauma and diagnosis of post-traumatic stress disorder (PTSD) is highly associated with both comorbid mood disorders [59] and migraine [43]. Given this background, it is perhaps not surprising that PTSD is more prevalent in patients with migraine than in the general population (14–25% vs. 1–12%), and is even more prevalent in patients with CM compared with patients with episodic migraine (43% vs. 9%), despite a similar frequency and prevalence of trauma exposure between the groups [43]. One study reported that 60% of migraine patients with PTSD had physical or sexual abuse as a traumatic life event and 42% of people with migraine reported physical or sexual assault [60]. In our study, the migraineurs in the mTBI group had significantly higher PHQ-2 scores and GAD-7 grades compared to the non-TBI group in initial analysis, which remained significant after adjusting for diagnosis of CM. Further, about a quarter of migraine patients in this sample reported having PTSD, with a significantly higher proportion of migraine patients in the mTBI group reported having PTSD compared to the non-TBI group. In summary, these findings are consistent with previous reports, and extend the body of data that support the complex relationship between affective symptom burden and migraine, migraine chronicity, and history of trauma.

A prior study found that among mTBI patients who were hospitalized, the most common reported cause of the injury was vehicle (55–58%), followed by fall (23–24%), and violence (5–10%) [61]. Meanwhile, another study found that mTBI outpatients who presented to a headache center reported that the cause of the injury was a fall (42–45%), followed by motor vehicle accident (18–24%) [36, 62]. These discrepancies in the cause of mTBI are explained by the severity of the injury, such as the tendency for traffic accidents to result in serious injuries compare to falls. Although the ARMR questionnaires did not ask directly about the cause of the TBI, the higher proportion of self-reported abuse history in the mTBI group than in the non-TBI group indicate that some of the TBIs could be due to abuse. TBI by abuse is more likely to be repetitive than TBI by motor vehicle accidents [63, 64]. Patients with mTBI suffer more PTH than their moderate-to-severe counterparts [65], and repeated TBI worsens the long-term prognosis of PTH [66]. While the nature of the reported physical abuse was not investigated in this study, it is notable that our sample was predominately female. Domestic violence occurs in approximately 1 in every 4 women in the United States, and up to 94% of injuries women sustain from abuse are to the neck and head [67–69]. Thus, given the prevalence of migraine in women, and the high rate of domestic violence and repetitive head injuries these women sustain, a history of domestic violence and physical abuse should be a part of the clinical evaluation of women with migraine. Moreover, future research should focus on the relationship between physical abuse, head injuries, and their impact on the clinical course of migraine.

### Limitations

In general, the strengths and limitations of the ARMR as a data source have been previously described [13]. In brief, since ARMR patients are enrolled from specialty headache centers, there is an overrepresentation of chronic migraine within the sample. Further, the overwhelming majority of participants within the sample are white, and thus results cannot necessarily be generalized to the overall US population. Additionally, the findings of this analyses should be interpreted as exploratory, given the potential for type I error inflation due to multiple comparisons.

Additionally, in the mTBI group, 145 patients reported “new onset or worsening headache within 7 days of their injury,” and thus may have a history consistent with the ICHD-based diagnosis of persistent PTH with a migraine or CM phenotype that was not coded by providers within the ARMR database. It is possible that when headaches have been present for a long time, clinicians may not ask about inciting/triggering events, such as mTBI, that led to worsening headache patterns, reflecting a systematic bias to overlook PTH when TBI is relatively remote or is overlaid on a history of migraine. In our sample, the effect of mTBI on migraine onset or headache worsening may be underestimated, since about 65% of the mTBI group reported a history of headaches before the most recent trauma reported in ARMR, and thus information about initial head injury and its relationship to headache onset was lacking. Most of the patients have a long history of headache and the TBI event occurred on median 14.4 years before enrollment into ARMR, making recall bias and recall error a limitation. The other limitation is that there is no detailed information on the frequency and intensity of headaches during the 14 years between the TBI event and the study enrollment. Future longitudinal studies are needed to better understand the natural history of migraine relative to recurrent TBI.

## Conclusion

About 38% of patients with migraine in our study had a history of mTBI, which occurred a median of over 14 years before enrollment. A history of mTBI was found to be associated with increased headache frequency, a diagnosis of chronic migraine, and greater headache-related disability, higher rates of cognitive dysfunction, reading/lack of sleep as a headache trigger, vertigo/dizziness, allodynia, and higher rates of anxiety and depression symptoms. The biological mechanisms underlying the increased prevalence of these features merit further exploration. A greater proportion of migraine patients with a history of mTBI reported a history of abuse, especially physical abuse. This study shows the necessity of inquiring about a lifetime history of TBI in patients being evaluated for migraine. People with migraine who have sustained prior TBI may represent a more severe phenotype and some patients with CM may actually represent persistent PTH with a CM phenotype. Since patients with persistent PTH are often clinically regarded as more resistant to conventional migraine treatments, these results raise the question as to whether this subgroup of patients, thought to have treatment-resistant CM, may actually have persistent PTH. This potential association will require further study.

### Key findings

● In the American Registry for Migraine Research patient population, 38% of patients with migraine had a history of mTBI.

● A history of mTBI is associated with increased diagnosis of chronic migraine, headache frequency, vertigo/dizziness, allodynia, reading/lack of sleep as a trigger, headache-related disability, cognitive dysfunction, anxiety, and depression.

● Symptoms eliciting a history of prior mTBI should be an integral part of the evaluation of patients with migraine, especially in those with a severe phenotype including chronic migraine.

● The extent to which a subgroup of patients diagnosed with CM in clinical practice actually has persistent PTH should be a focus of future research.

## Data Availability

The datasets used and/or analysed during the study ae available from the corresponding author with approval of the ARMR on reasonable request.

## Abbreviations

ARMR: The American registry for migraine research
ASC-12: Allodynia symptom checklist-12
CM: Chronic migraine
GAD-7: General anxiety disorder-7
ICHD-3: The international classification of headache disorders 3rd edition
IQR: Interquartile range
MIDAS: Migraine disability assessment scale
MIG-SCog: Subjective cognitive impairment during migraine attacks
MOH: Medication-overuse headache
mTBI: Mild traumatic brain injury
MwA: Migraine with aura
NRS: Numeric rating scale
PHQ-2: Patient health questionnaire-2
PROMIS-PI: Patient-reported outcomes measurement information system- pain Interference
PTH: Post-traumatic headache
PTSD: Post-traumatic stress disorder
TBI: Traumatic brain injury
WPAI: Work productivity and activity impairment due to headache
